# Impact of preoperative venovenous extracorporeal membrane oxygenation bridging on postoperative acute kidney injury after lung transplantation

**DOI:** 10.1007/s10047-026-01585-4

**Published:** 2026-08-17

**Authors:** Rachel Reichenbach, Yudai Miyashita, Taichi Nagano, Taisuke Kaiho, Yuriko Yagi, Ankit Bharat, G. R. Scott Budinger, Ambalavanan Arunachalam, Chitaru Kurihara

**Affiliations:** 1 Division of Thoracic Surgery, Department of Surgery, Northwestern University Feinberg School of Medicine, Chicago, IL 60611 USA; 2 Division of Pulmonary and Critical Care Medicine, Department of Medicine, Northwestern University Feinberg School of Medicine, Chicago, IL USA; 3 Division of Thoracic Surgery, Northwestern University Feinberg School of Medicine, 60611, 676 N. Saint Clair St., Suite 650, IL Chicago, USA

**Keywords:** Lung transplantation, Venovenous extracorporeal membrane oxygenation (VV-ECMO) bridge, Acute kidney injury (AKI)

## Abstract

Venovenous extracorporeal membrane oxygenation (VV-ECMO) bridging to lung transplant has the potential to reduce mortality among patients awaiting transplant. However, there continue to be notable morbidity risks, including acute kidney injury (AKI). This paper seeks to evaluate both short and long-term morbidity and mortality trends related to AKI development post-transplant, among VV-ECMO bridged and non-ECMO bridged patients respectively. We performed a retrospective cohort study of adult patients (≥ 18 years) who underwent lung transplantation at a single tertiary care center between January 2018 and May 2024. 443 lung transplant recipients were included: 46 were bridged with preoperative VV-ECMO, and 397 underwent transplantation without ECMO bridge. Univariate logistic regression was used to evaluate predictors of postoperative AKI. A multivariable logistic regression model was then constructed using clinically relevant covariates and variables associated with AKI in univariate analyses. The initial incidence of post-operative AKI was significantly higher among patients bridged with VV-ECMO (67% vs. 44% *P* = 0.005), as were overall complications, including primary graft dysfunction grade 3 (41% vs. 9.8%, *P* < 0.001) and longer duration of mechanical ventilation (6.0 [2.0–17.0] vs. 2.0 [1.0–3.0] days, *P* < 0.001). However, there was no significant difference in renal function trajectories between VV-ECMO bridged and non-bridged groups from 1 month to 1 year, operationalized via GFR and creatinine trends. There was no statistically significant difference in survival between VV-ECMO bridged and non-bridged groups (*P* = 0.93). Independent of bridging status, patients who developed AKI post-operatively had significantly worse short and long-term survival after transplantation than those without AKI (log-rank *P* < 0.0001). Preoperative VV-ECMO bridging was not associated with a statistically significant difference in overall survival during the available follow-up. In contrast, postoperative AKI was independently associated with increased mortality after lung transplantation.

**Supplementary Information:**

The online version contains supplementary material available at https://doi.org/10.1007/s10047-026-01585-4.

## Introduction

Venovenous extracorporeal membrane oxygenation (VV-ECMO) bridging to lung transplant has been a key area of growth in the field of thoracic transplant over the last several decades. VV-ECMO bridging offers a unique modality of aiding some of our most critically ill patients in successfully arriving at definitive treatment: lung transplant. Existing literature has described the evolving, and improving, outcome profile of VV-ECMO bridging among patients awaiting organ availability, particularly when treated at high volume, experienced centers [1, 2]. However, despite the positive potential of VV-ECMO bridging to reduce mortality among patients awaiting transplant, there continue to be notable morbidity risks [2–4]. One of the common post-operative complications, both among patients requiring ECMO, as well as patients undergoing lung transplant, is acute kidney injury (AKI). There are multiple known risk factors for kidney injury in the lung transplant population specifically, including nephrotoxic medications and peri-operative ECMO use [5, 6]. There is some debate as to the degree to which post-transplant AKI is causal versus associational in its connection to peri-transplant ECMO use. Existing literature has broadly described the occurrence of AKI in patients requiring ECMO for any reason, up to 63% in one meta-analysis [6–9]. However, lung transplant itself is also thought to have unique links to the development of AKI, involving mechanisms involving ischemia-reperfusion injury, as well as primary graft dysfunction [6, 10]. Despite existing literature describing the incidence of renal injury, little research has directly evaluated renal injury both in relation to, and independently from, patients’ VV-ECMO bridging status. A single-center retrospective study by Gao et al. [5] found that AKI in ECMO patients was not associated with increased mortality; however, they had no non-ECMO group for comparison. Another study by Rando et al. [2], only examined ECMO as a broad category, without breakdown into VV vs. VA-ECMO, an important distinguishing factor to assess when looking at outcomes. Therefore, the present study seeks to address these current gaps in literature by evaluating short and long-term morbidity and mortality trends according to AKI development post lung transplant, among VV-ECMO bridged and non-ECMO bridged transplant patients respectively.

## Methodology

### Study design

This was a retrospective cohort study of adult patients (≥ 18 years) who underwent lung transplantation at a single tertiary care center between January 2018 and May 2024. Clinical data were obtained from the electronic medical record and the institutional lung transplant database at Northwestern Memorial Hospital (Chicago, Illinois, USA). Multi-organ transplants and redo lung transplant procedures were excluded; only primary isolated lung transplants were included in the analytic cohort. The study protocol was approved by the Institutional Review Board of Northwestern University (STU00212120). The requirement for informed consent for data collection and analysis was waived because of the retrospective nature of the study. All transplants were performed according to institutional protocols, United States regulations, the Declaration of Helsinki, and the principles of the Declaration of Istanbul on Organ Trafficking and Transplant Tourism; no organs were procured from prisoners or other unethical sources. Peri-operative and post-operative management at our center has been described previously [11–14]. Detailed definitions of all study variables and complications are provided in the Supplemental Materials.

### Immunosuppression management

Induction

- Methylprednisolone 1000 mg IV intraoperatively.
- Basiliximab 20 mg intraoperatively and POD 4.

Maintenance immunosuppression

- Prednisone 0.5 mg/kg P.O. daily from POD 1. Maximum dose = prednisone 40 mg daily. 0.5 mg/kg daily for 1 month, then taper by 5 mg every 2 weeks down to 5 mg/day as a maintenance dose.
- Mycophenolate mofetil 1,000 mg B.I.D. from POD 1.
- Tacrolimus starting POD 1. Goal target levels 8–12 within first year post-transplant; then target 8–10 thereafter.

### VV-ECMO indication criteria

Prior to lung transplantation, all intubated patients were treated by a multidisciplinary team in accordance with the guidelines of the National Heart, Lung, and Blood Institute’s ARDS Network [15]. Indications for ECMO evaluation included refractory hypoxemia with PaO_2_ less than 55 mmHg, pulse oximetry oxygen saturation less than 88%, and pH level less than 7.2. Patients were evaluated with lung-protective mechanical ventilation with a plateau pressure of less than 35 mmHg, neuromuscular blockade, and prone positioning, according to recommendations from the Extracorporeal Life Support Organization [16].

### Intraoperative VA-ECMO indications

We routinely employ VA-ECMO during lung transplantation to capitalize on several key advantages that enhance surgical and postoperative outcomes [17–22]. Specifically, VA-ECMO is indicated in these settings when specific factors require further optimization. Specifically, VA-ECMO aids in the following:

- Maintaining hemodynamic stability, especially when surgical access to the hilar structures is challenging.
- Facilitating the use of lung-protective ventilation strategies by allowing for low inspired oxygen fractions and reduced driving pressures.
- Allowing for prolonged, controlled reperfusion of the newly implanted graft.
- Improving right heart support by minimizing right ventricular strain, particularly during pulmonary artery clamping.

### Infection prophylaxis and CMV monitoring strategies

Pneumocystis jirovecii pneumonia prophylaxis was uniformly administered using Bactrim DS twice weekly (Mondays and Wednesdays). CMV prophylaxis was tailored based on the serostatus risk stratification of donors and recipients. Specifically, patients with CMV R(+) D(+) and R(+) D(–) status received valganciclovir 900 mg orally once daily for 6 months, while those with CMV R(–) D(+) status were treated for 12 months. In selected cases of CMV R(+) D(+) patients, a shorter regimen of valacyclovir (500 mg orally twice daily for 3 months) was employed depending on individual risk factors. Fungal prophylaxis was ensured with posaconazole administered for 6 months, and in patients receiving eculizumab, meningococcal prophylaxis with Penicillin V for 3 months was instituted. Furthermore, all patients–regardless of sensitization status–received vaccination against meningococcal disease ideally at least 2 weeks prior to transplant listing: a measure particularly vital for sensitized patients on eculizumab to mitigate the risk of meningococcal infection. Concurrent with these pharmacological interventions, our center implemented an intensive CMV surveillance protocol. Quantitative CMV PCR monitoring was performed every 1–2 weeks during the first three months post-transplant, then reduced to 2–4 weeks between months 3 and 12. More specifically, the schedule involved weekly measurements for the first 4 weeks, biweekly assessments during the second month, monthly monitoring over the following 9 months, and subsequent evaluations every three months. After discontinuation of valganciclovir at one-year, biweekly PCR testing was carried out for an additional month, with further monitoring reserved for symptomatic patients.

### Definition of complications

#### Primary graft dysfunction (PGD)

PGD was defined based on the ISHLT guidelines [23], requiring the presence of bilateral pulmonary infiltrates on chest radiograph. Grading was based on the PaO2/FiO2 ratio as follows; Grade 1: PaO2/FiO2 ratio > 300; Grade 2: PaO2/FiO2 ratio is 200–300; Grade 3: PaO2/FiO2 ratio < 200 or use of ECMO for hypoxemia. For assessments performed within 72 h, a more severe threshold was incorporated.

#### Acute kidney injury (AKI)

Postoperative AKI was identified during the index transplant hospitalization and classified according to the Risk, Injury, Failure, Loss of kidney function, and End-stage kidney disease(RIFLE)criteria [24]. The preoperative dialysis variable indicated receipt of renal replacement therapy before transplantation and included patients receiving temporary renal replacement therapy for acute critical illness. A history of preoperative dialysis alone was not considered sufficient to define postoperative AKI. In these patients, postoperative AKI status was determined based on the documented postoperative renal course, including worsening or persistent renal dysfunction and the postoperative requirement for renal replacement therapy relative to the pretransplant clinical status.

#### Respiratory infections

Defined as the presence of clinical symptoms (fever, increased cough, purulent sputum, dyspnea) combined with radiographic evidence of new or progressive infiltrates, and confirmed by microbiological testing (e.g., sputum culture, bronchoalveolar lavage) [25].

#### Chronic lung allograft dysfunction (CLAD)

CLAD is defined as a sustained (at least 3 months) decline in the forced expiratory volume in one second (FEV₁) of at least 20% from the post-transplant baseline, in the absence of other reversible causes. CLAD is further sub-classified into phenotypes such as bronchiolitis obliterans syndrome (BOS) and restrictive allograft syndrome (RAS) based on clinical, radiologic, and physiologic criteria [26].

### Statistical analysis

Clinical characteristics and perioperative outcomes were compared between patients with and without preoperative VV-ECMO bridging using the Wilcoxon rank-sum test for continuous variables and Fisher’s exact test for categorical variables, as appropriate. Continuous variables are expressed as medians (IQRs) and categorical variables as numbers (%). Postoperative outcomes included de novo donor-specific antibody formation, cerebrovascular accident, bowel ischemia, digital ischemia, pulmonary embolism, AKI, PGD3, postoperative dialysis, hemodialysis after discharge, post-transplant ventilator days, hospital length of stay, and postoperative ECMO use. Univariate logistic regression was used to evaluate predictors of postoperative AKI. A multivariable logistic regression model was then constructed using clinically relevant covariates and variables associated with AKI in univariate analyses. The primary multivariable logistic regression model was restricted to variables available before transplantation because the primary clinical objective was to evaluate whether postoperative AKI risk could be identified preoperatively. This model included preoperative VV-ECMO bridging, age, sex, smoking history, hypertension, diabetes, underlying lung disease etiology, hemoglobin, and serum creatinine.

In response to the potential influence of operative complexity, an additional explanatory model was constructed that further included bilateral transplantation, operative time, and intraoperative packed red blood cell transfusion. Packed red blood cell transfusion was selected as a representative measure of transfusion burden to avoid simultaneously including multiple closely related blood-product variables. Because intraoperative factors occur after preoperative VV-ECMO exposure and may lie on the causal pathway between bridging status and AKI, this expanded model was considered an explanatory analysis rather than the primary preoperative prediction model. As a sensitivity analysis, propensity score overlap weighting was performed to further address differences in pretransplant characteristics between patients with and without VV-ECMO bridging. Propensity scores were estimated using a logistic regression model that included age, sex, body mass index, smoking history, hypertension, diabetes, chronic kidney disease, preoperative dialysis, waiting-list duration, underlying lung disease etiology, hemoglobin, platelet count, serum creatinine, and panel reactive antibody status. Intraoperative variables were not included in the propensity score model because they occurred after preoperative VV-ECMO exposure and could represent mediators of the association with postoperative AKI. Overlap weights were assigned as one minus the propensity score for VV-ECMO-bridged patients and as the propensity score for non-ECMO-bridged patients. Covariate balance was evaluated using absolute standardized mean differences, with values below 0.10 considered indicative of adequate balance. The association between preoperative VV-ECMO bridging and postoperative AKI was subsequently evaluated using overlap-weighted logistic regression. Results are reported as odds ratios with 95% confidence intervals. Longitudinal renal function was assessed using serum creatinine and eGFR at baseline and at 1, 3, 6, and 12 months after transplantation. Comparisons between groups at each time point were performed using the Wilcoxon rank-sum test. Overall survival was analyzed using the Kaplan-Meier method and compared with the log-rank test according to preoperative VV-ECMO bridge status and postoperative AKI status. All statistical tests were two-sided, and *P* < 0.05 was considered statistically significant. Analyses were performed using R (R Foundation for Statistical Computing, Vienna, Austria).

## Results

### Patient characteristics

Among 443 lung transplant recipients included in the study, 46 patients were bridged with preoperative VV-ECMO, whereas 397 underwent transplantation without ECMO bridge (Table 1). Patients in the VV-ECMO bridge group were significantly younger than those in the non-ECMO bridge group (53.0 [35.0–58.0] vs. 63.0 [56.0–69.0] years, *P* < 0.001). Smoking history was less frequent in the VV-ECMO bridge group (22% vs. 54%, *P* < 0.001), while preoperative dialysis was more common (15% vs. 3.8%, *P* = 0.004). Patients with VV-ECMO bridging also had a shorter waiting time before transplantation (5.5 [4.0–21.0] vs. 11.0 [5.0–34.0] days, *P* = 0.02).

**Table 1:** Characteristics of patients

Variable	VV ECMO bridge (*n* = 46)	No ECMO bridge (*n* = 397)	*P* value
Pre-operative characteristics			
Age, years	53.0 (35.0–58.0)	63.0 (56.0–69.0)	< 0.001
Sex			0.43
Male	23 (50%)	226 (57%)	
Female	23 (50%)	171 (43%)	
BMI, kg/m^2^	26.6 (22.8–29.0)	26.5 (22.2–29.6)	0.77
BSA, m^2^	1.9 (1.7–2.0)	1.9 (1.7-2.0)	0.83
Smoking history	10 (22%)	215 (54%)	< 0.001
Hypertension	20 (43%)	235 (59%)	0.06
Diabetes	12 (26%)	124 (31%)	0.61
CKD	3 (6.5%)	39 (9.8%)	0.60
Dialysis	7 (15%)	15 (3.8%)	0.004
On the waiting list (days)	5.5 (4.0–21.0)	11.0 (5.0–34.0)	0.02
ECMO bridge			
Etiology			< 0.001
ILD	11 (24%)	202 (51%)	
COPD	0 (0%)	87 (22%)	
PAH	3 (6.5%)	41 (10%)	
Others	32 (70%)	67 (17%)	
Laboratory			
Hemoglobin, g/dL	8.1 (7.2–8.5)	12.0 (10.7–13.6)	< 0.001
Platelets, 1000/mm^3^	152.5 (122.0–221.0)	243.5 (200.0-304.0)	< 0.001
Creatinine, mg/dL	0.6 (0.4–0.8)	0.8 (0.6–0.9)	< 0.001
eGFR	116.5 (102.5-126.5)	95.7 (82.2-104.2)	< 0.001
INR	1.2 (1.1–1.3)	1.0 (1.0-1.1)	< 0.001
PTT	34.3 (30.5–39.5)	30.4 (27.8–33.0)	< 0.001
PRA	20 (43%)	249 (63%)	0.02
Intra-operative outcomes			
Bilateral	45 (98%)	232 (58%)	< 0.001
Operative time (hours)	8.3 (7.1–9.7)	5.4 (4.2–6.9)	< 0.001
Intra-op blood transfusion; pRBC	8.5 (6.0–14.0)	0.0 (0.0–2.0)	< 0.001
Intra-op blood transfusion; FFP	2.5 (1.0–6.0)	0.0 (0.0–0.0)	< 0.001
Intra-op blood transfusion; Plt	2.0 (1.0–4.0)	0.0 (0.0–0.0)	< 0.001
VA ECMO use	43 (93%)	225 (57%)	< 0.001

Continuous variables are presented as median (interquartile range), and categorical variables are presented as number (%). P values were calculated using the Wilcoxon rank-sum test for continuous variables and Fisher’s exact test for categorical variables. VV-ECMO, venovenous extracorporeal membrane oxygenation; BMI, body mass index; BSA, body surface area; CKD, chronic kidney disease; ILD, interstitial lung disease; COPD, chronic obstructive pulmonary disease; PAH, pulmonary arterial hypertension; eGFR, estimated glomerular filtration rate; INR, international normalized ratio; PTT, partial thromboplastin time; PRA, panel reactive antibody; pRBC, packed red blood cells; FFP, fresh frozen plasma; Plt, platelets; VA-ECMO, venoarterial extracorporeal membrane oxygenation.

The distribution of transplant etiologies differed significantly between groups (*P* < 0.001). Interstitial lung disease was less common in the VV-ECMO bridge group (24% vs. 51%), whereas the proportion categorized as other etiologies was substantially higher (70% vs. 17%). Preoperative laboratory profiles also differed significantly. Compared with patients without ECMO bridging, those with VV-ECMO bridging had lower hemoglobin (8.1 [7.2–8.5] vs. 12.0 [10.7–13.6] g/dL, *P* < 0.001), lower platelet counts (152.5 [122.0–221.0] vs. 243.5 [200.0–304.0] ×10^3/mm^3, *P* < 0.001), lower creatinine (0.6 [0.4–0.8] vs. 0.8 [0.6–0.9] mg/dL, *P* < 0.001), higher eGFR (116.5 [102.5–126.5] vs. 95.7 [82.2–104.2], *P* < 0.001), higher INR (1.2 [1.1–1.3] vs. 1.0 [1.0–1.1], *P* < 0.001), higher PTT (34.3 [30.5–39.5] vs. 30.4 [27.8–33.0], *P* < 0.001), and a lower prevalence of positive PRA (43% vs. 63%, *P* = 0.02).

Intraoperatively, patients bridged with VV-ECMO underwent a more complex operative course. Bilateral transplantation was performed more frequently in the VV-ECMO bridge group (98% vs. 58%, *P* < 0.001), operative time was longer (8.3 [7.1–9.7] vs. 5.4 [4.2–6.9] hours, *P* < 0.001), and intraoperative transfusion requirements were markedly greater for packed red blood cells, fresh frozen plasma, and platelets (all *P* < 0.001). Intraoperative VA-ECMO use was also more frequent in the VV-ECMO bridge group (93% vs. 57%, *P* < 0.001).

### Postoperative outcomes

**Fig. 1:**
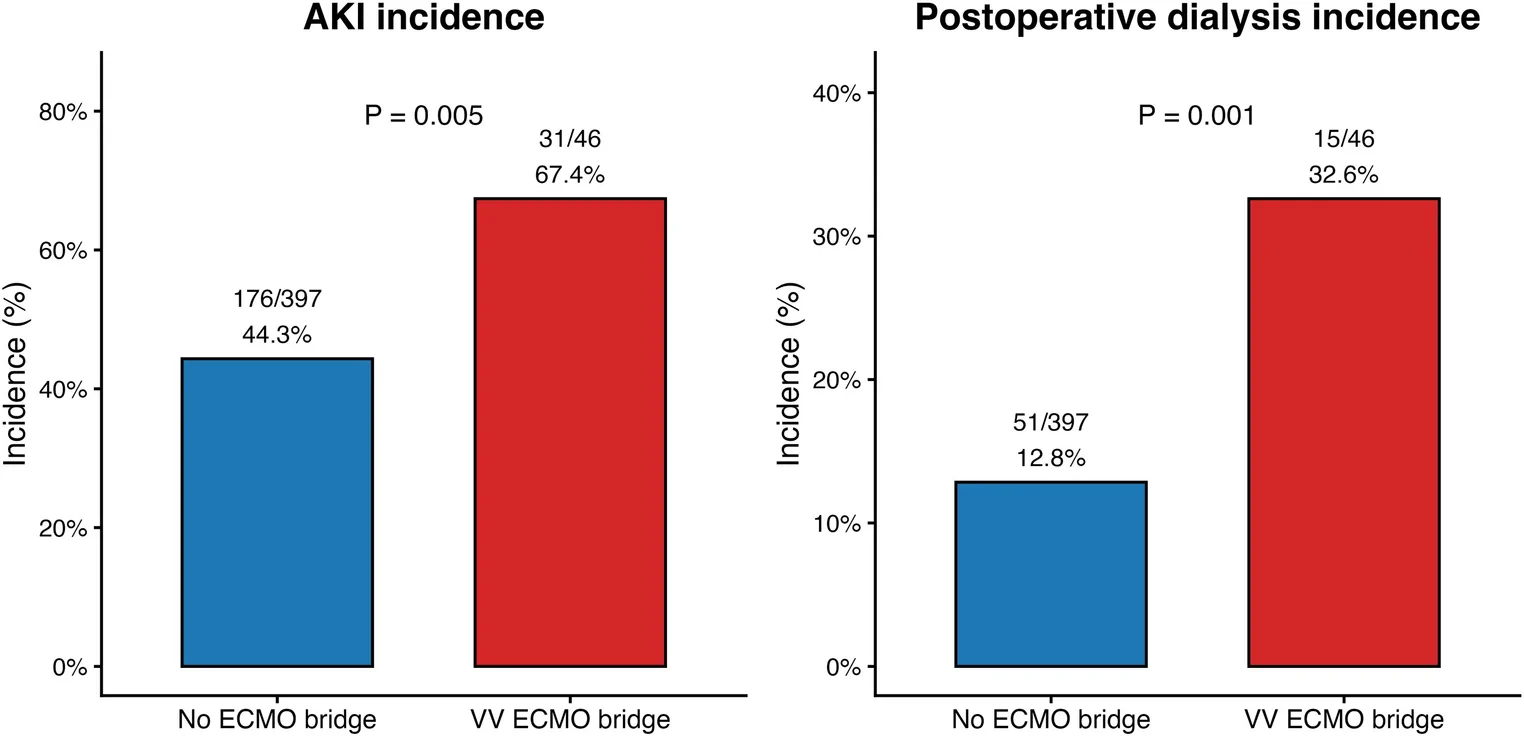
Incidence of postoperative acute kidney injury and dialysis according to preoperative VV-ECMO bridging status.The incidence of postoperative acute kidney injury (AKI) and postoperative dialysis was compared between patients with and without preoperative venovenous extracorporeal membrane oxygenation (VV-ECMO) bridging. Numbers above the bars indicate the number of events/total number of patients and percentages. P values were calculated using Fisher’s exact test

Postoperative outcomes differed substantially between groups (Table 2). The incidence of postoperative acute kidney injury (AKI) was significantly higher among patients bridged with VV-ECMO than among those without ECMO bridge (67% vs. 44%, *P* = 0.005), and the need for postoperative dialysis was also more frequent (33% vs. 13%, *P* = 0.001). Similarly, the incidence of post-discharge hemodialysis remained significantly higher in the VV-ECMO bridge group (26% vs. 12%, *P* = 0.01) (Fig. 1).

**Table 2:** Post-operative outcomes

Variable	VV ECMO bridge (*n* = 46)	No ECMO bridge (*n* = 397)	*P* value
Post-operative outcomes			
De novo DSA	11 (24%)	61 (15%)	0.14
CVA	1 (2.2%)	14 (3.5%)	1.00
Bowel ischemia	1 (2.2%)	5 (1.3%)	0.48
Digital ischemia	3 (6.5%)	5 (1.3%)	0.04
PE	7 (15%)	55 (14%)	0.82
AKI	31 (67%)	176 (44%)	0.005
PGD grade3	19 (41%)	39 (9.8%)	< 0.001
Dialysis	15 (33%)	51 (13%)	0.001
HD after discharge	12 (26%)	46 (12%)	0.01
Post transplant ventilator (days)	6.0 (2.0–17.0)	2.0 (1.0–3.0)	< 0.001
Hospital stay (days)	36.5 (24.0–47.0)	16.0 (11.0–29.0)	< 0.001
Post ECMO use	30 (65%)	27 (6.8%)	< 0.001

Continuous variables are presented as median (interquartile range), and categorical variables are presented as number (%). P values were calculated using the Wilcoxon rank-sum test for continuous variables and Fisher’s exact test for categorical variables. DSA, donor-specific antibody; CVA, cerebrovascular accident; PE, pulmonary embolism; AKI, acute kidney injury; PGD, primary graft dysfunction; HD, hemodialysis; ECMO, extracorporeal membrane oxygenation.

In addition to renal complications, patients with preoperative VV-ECMO bridge experienced a higher rate of PGD grade 3 (41% vs. 9.8%, *P* < 0.001), longer duration of post-transplant mechanical ventilation (6.0 [2.0–17.0] vs. 2.0 [1.0–3.0] days, *P* < 0.001), longer hospital stay (36.5 [24.0–47.0] vs. 16.0 [11.0–29.0] days, *P* < 0.001), and more frequent postoperative ECMO use (65% vs. 6.8%, *P* < 0.001). Digital ischemia was also more common in the VV-ECMO bridge group (6.5% vs. 1.3%, *P* = 0.04). In contrast, no statistically significant between-group differences were observed in de novo donor-specific antibody formation, cerebrovascular accident, bowel ischemia, or pulmonary embolism.

### Risk factors for AKI

**Fig. 2:**
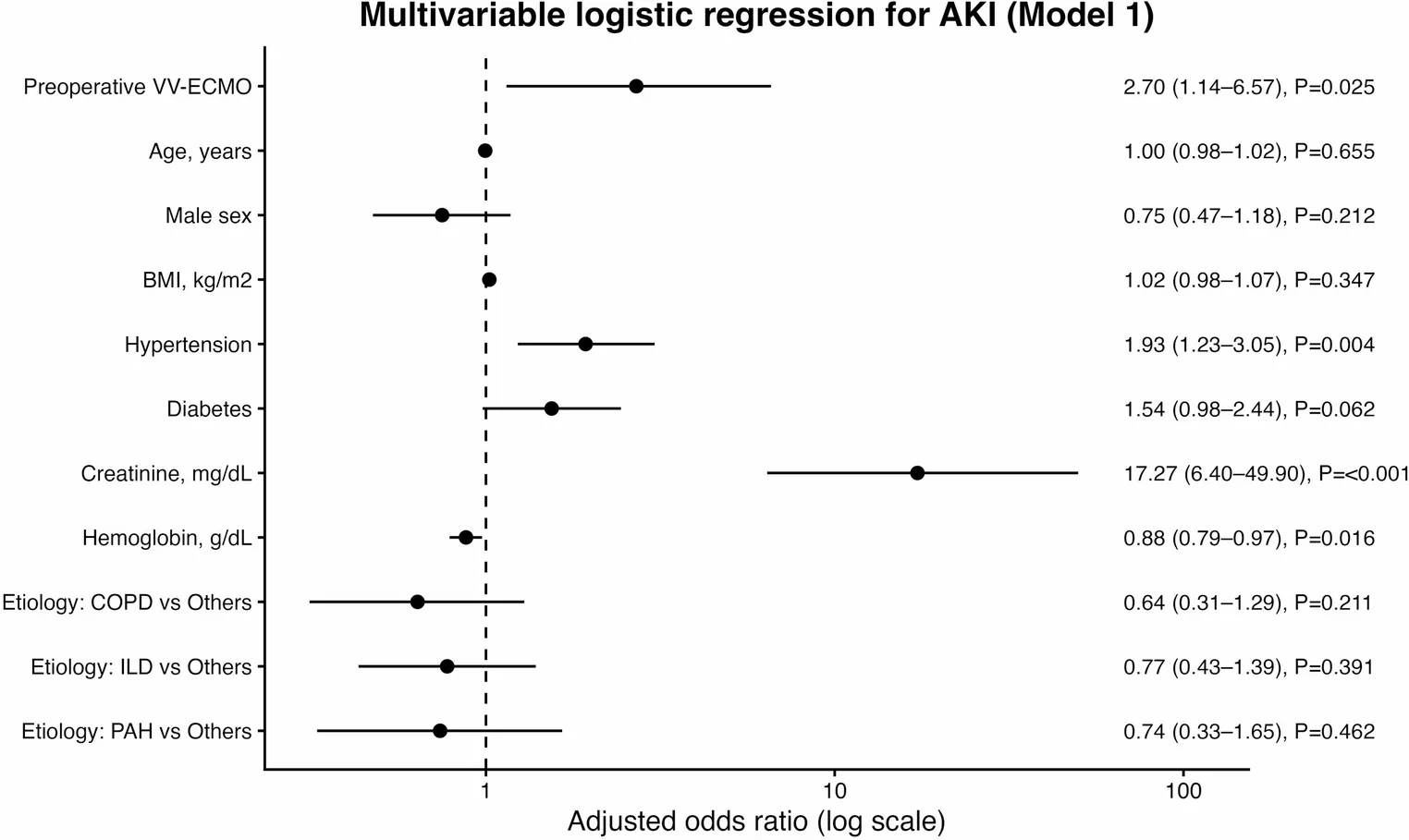
Multivariable logistic regression analysis of predictors of postoperative acute kidney injury.Forest plot showing adjusted odds ratios and 95% confidence intervals for factors associated with postoperative acute kidney injury (AKI) in the primary multivariable model. The dashed vertical line indicates an odds ratio of 1. VV-ECMO, venovenous extracorporeal membrane oxygenation; BMI, body mass index; COPD, chronic obstructive pulmonary disease; ILD, interstitial lung disease; PAH, pulmonary arterial hypertension

To identify factors associated with postoperative AKI, we performed univariate logistic regression analysis. Higher BMI (OR 1.05, 95% CI 1.01–1.10, *P* = 0.01), hypertension (OR 1.96, 95% CI 1.33–2.88, *P* < 0.001), diabetes (OR 1.78, 95% CI 1.18–2.68, *P* = 0.006), chronic kidney disease (OR 4.13, 95% CI 2.05–9.07, *P* < 0.001), preoperative dialysis (OR 5.52, 95% CI 2.02–19.37, *P* = 0.002), preoperative VV-ECMO bridge (OR 2.60, 95% CI 1.38–5.08, *P* = 0.004), lower hemoglobin (OR 0.89, 95% CI 0.82–0.96, *P* = 0.004), and higher creatinine (OR 7.10, 95% CI 3.21–16.44, *P* < 0.001) were significantly associated with postoperative AKI. Age, smoking history, waiting list duration, etiology of lung, platelet count, INR, PTT, and PRA were not significantly associated with AKI (Table 3). In our multivariable logistic regression, preoperative VV-ECMO bridge remained independently associated with postoperative AKI (adjusted OR 2.70, 95% CI 1.14–6.57, *P* = 0.025). Hypertension (adjusted OR 1.93, 95% CI 1.23–3.05, *P* = 0.004) and higher creatinine (adjusted OR 17.27, 95% CI 6.40–49.90, *P* < 0.001) were also independently associated with increased odds of AKI, whereas higher hemoglobin was associated with lower odds of AKI (adjusted OR 0.88, 95% CI 0.79–0.97, *P* = 0.016). Age, sex, BMI, diabetes, and underlying disease etiology were not independently associated with postoperative AKI in the adjusted model (Fig. 2). Because patients bridged with VV-ECMO experienced substantially greater operative complexity, we constructed an additional model including bilateral transplantation, operative time, and intraoperative packed red blood cell transfusion. After adjustment for these intraoperative variables, the association between preoperative VV-ECMO bridging and postoperative AKI was attenuated and was no longer statistically significant. Greater intraoperative packed red blood cell transfusion remained independently associated with postoperative AKI, whereas bilateral transplantation and operative time were not independently associated with AKI (Supplemental Table 1).

**Table 3:** Univariate logistic regression analysis for predictors of postoperative AKI

	Univariate		
Variable	OR	95% CI	P value
Pre-operative characteristics			
Age, years	1.00	0.98–1.01	0.81
Sex			
Male	0.99	0.68–1.44	0.95
BMI, kg/m^2^	1.05	1.01–1.10	0.01
BSA, m^2^	2.15	1.00-4.65	0.05
Smoking history	1.33	0.92–1.94	0.13
Hypertension	1.96	1.33–2.88	< 0.001
Diabetes	1.78	1.18–2.68	0.006
CKD	4.13	2.05–9.07	< 0.001
Dialysis	5.52	2.02–19.37	0.002
On the waiting list (days)	1.00	0.99-1.00	0.33
Preoperative VV-ECMO	2.60	1.38–5.08	0.004
Etiology			
ILD	0.76	0.47–1.22	0.25
COPD	0.67	0.37–1.19	0.17
PAH	0.83	0.40–1.68	0.60
Laboratory			
Hemoglobin, g/dL	0.89	0.82–0.96	0.004
Platelets, 1000/mm^3^	1.00	1.00–1.00	0.21
Creatinine, mg/dL	7.10	3.21–16.44	< 0.001
INR	1.31	0.48–3.73	0.60
PTT	0.99	0.98–1.01	0.44
PRA	0.92	0.62–1.34	0.65

Odds ratios (ORs) and 95% confidence intervals (CIs) were estimated using univariate logistic regression models for postoperative AKI. VV-ECMO, venovenous extracorporeal membrane oxygenation; BMI, body mass index; BSA, body surface area; CKD, chronic kidney disease; ILD, interstitial lung disease; COPD, chronic obstructive pulmonary disease; PAH, pulmonary arterial hypertension; INR, international normalized ratio; PTT, partial thromboplastin time; PRA, panel reactive antibody; AKI, acute kidney injury; OR, odds ratio; CI, confidence interval.

In the propensity score overlap-weighting sensitivity analysis, substantial preoperative imbalances were observed before weighting, particularly in age, underlying lung disease etiology, hemoglobin, platelet count, and serum creatinine. After overlap weighting, all measured preoperative covariates achieved adequate balance, with absolute standardized mean differences below 0.10 (Supplemental Table 2). In the overlap-weighted logistic regression analysis, preoperative VV-ECMO bridging was associated with an increased point estimate for postoperative AKI, although the association did not reach statistical significance (weighted OR 2.34, 95% CI 0.87–6.31, *P* = 0.093; Supplemental Table 3).

### Renal function trajectories after transplantation

**Fig. 3:**
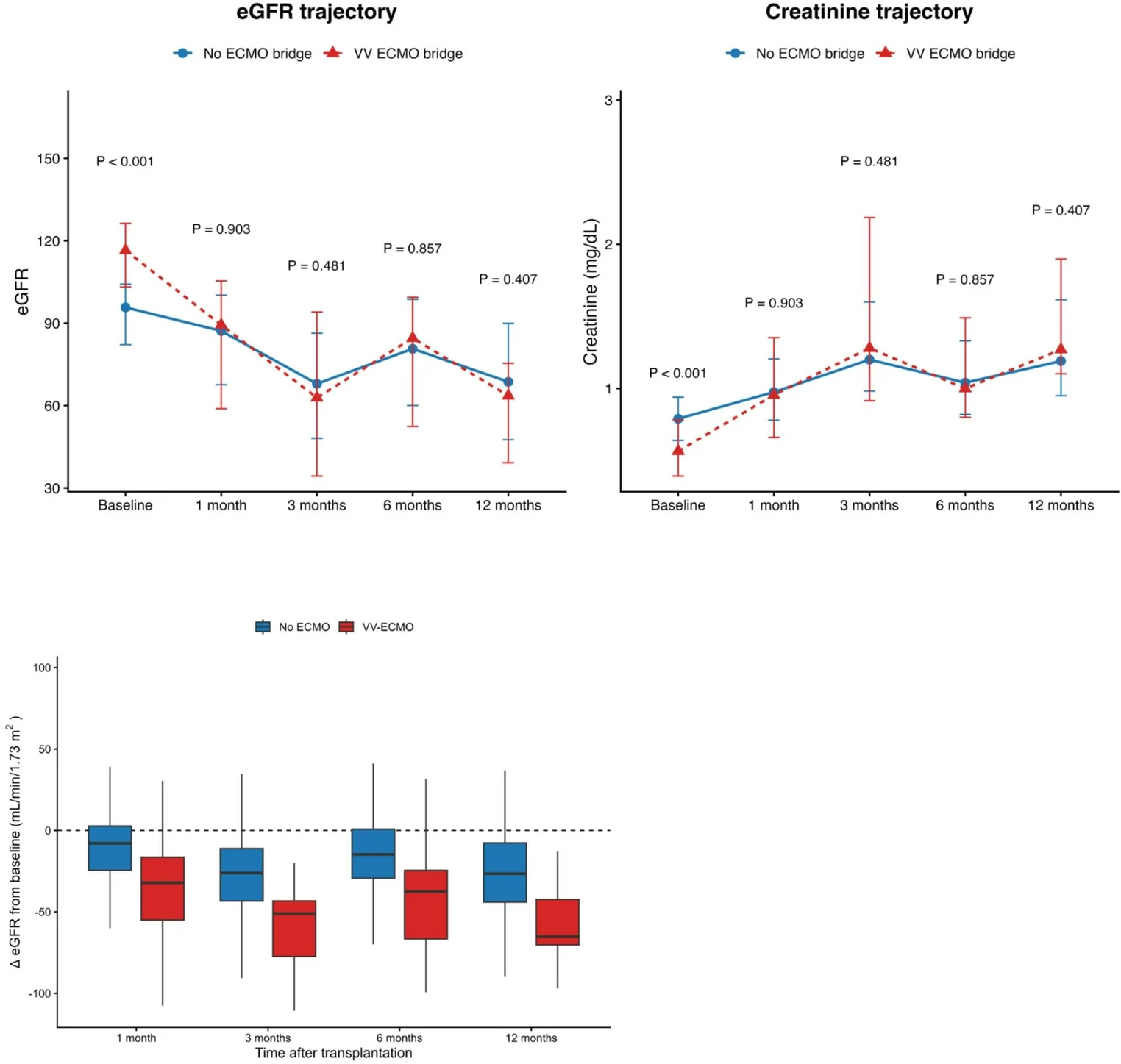
Longitudinal renal function after lung transplantation according to preoperative VV-ECMO bridging status.Estimated glomerular filtration rate (eGFR) and serum creatinine were compared between patients with and without preoperative venovenous extracorporeal membrane oxygenation (VV-ECMO) bridging at baseline and at 1, 3, 6, and 12 months after transplantation. The lower panel shows changes in eGFR from baseline at each post-transplant time point. P values represent between-group comparisons at each time point

We next examined longitudinal renal function after transplantation (Fig. 3). Patients bridged with preoperative VV-ECMO had significantly different baseline renal profiles, with higher eGFR and lower creatinine before transplantation. However, after transplantation, eGFR and creatinine trajectories were not significantly different between the VV-ECMO bridge and no-ECMO bridge groups at 1, 3, 6, or 12 months, suggesting that the early postoperative difference was not sustained over time. Specifically, between-group differences were significant only at baseline for both eGFR and creatinine (*P* < 0.001), whereas all subsequent time points were not statistically significant. In contrast, when stratified by postoperative AKI status, renal dysfunction persisted throughout follow-up. Patients who developed AKI had significantly lower eGFR and higher creatinine at baseline, 1 month, 3 months, 6 months, and 12 months after transplantation compared with those who did not develop AKI (all *P* < 0.001) (Supplemental Fig. 1).

### Association of VV ECMO use with overall survival

**Fig. 4:**
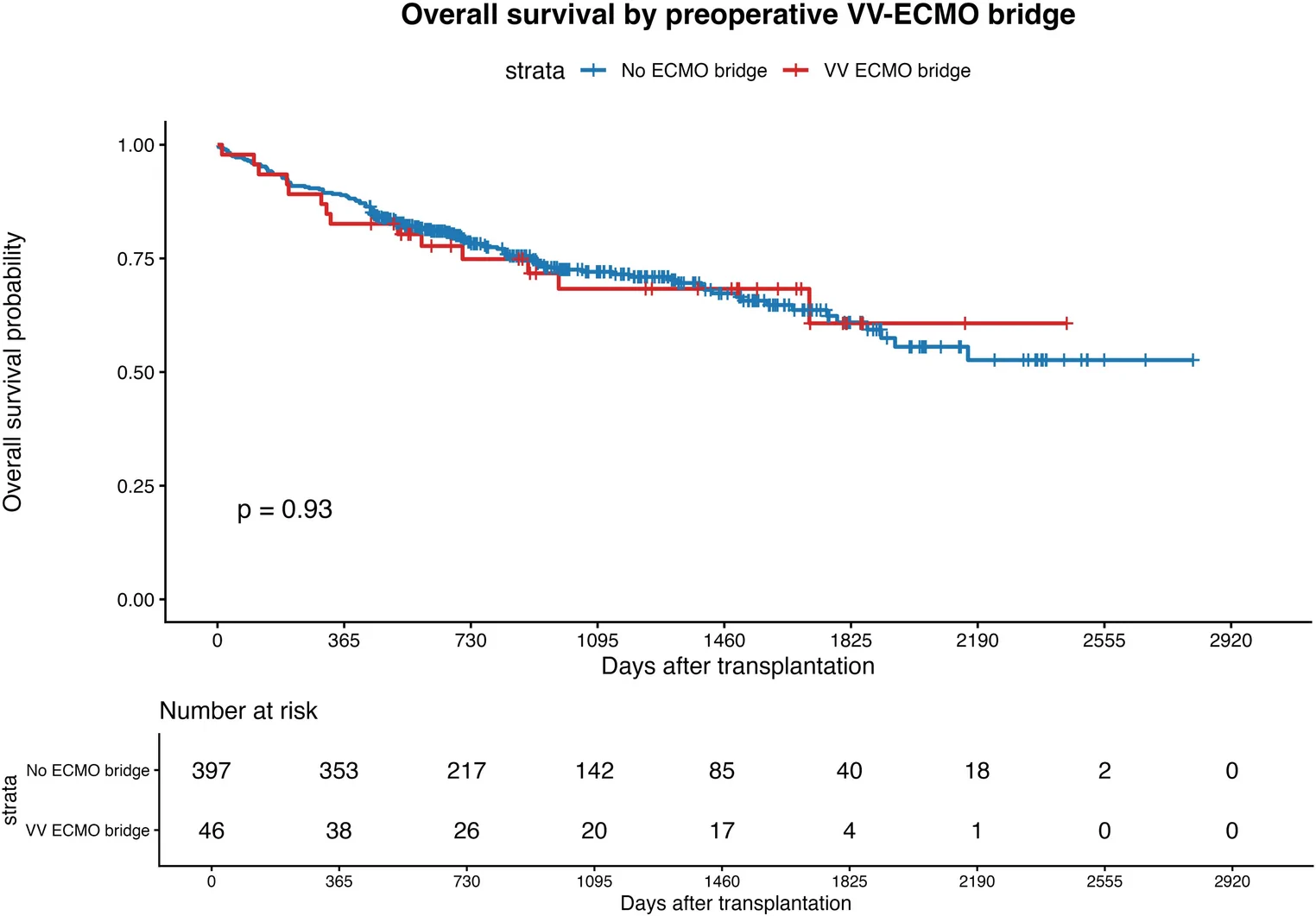
Overall survival after lung transplantation according to preoperative VV-ECMO bridging status.Kaplan–Meier curves show overall survival after lung transplantation in patients with and without preoperative venovenous extracorporeal membrane oxygenation (VV-ECMO) bridging. Survival did not significantly differ between the groups (log-rank P=0.93). Numbers at risk are shown below the survival curves

The median follow-up duration, estimated using the reverse Kaplan–Meier method, was 1112 days(3.0 years). At 5 years after transplantation, 44 patients remained at risk.　Kaplan-Meier analysis showed that overall survival did not significantly differ according to preoperative VV-ECMO bridge status (*P* = 0.93), despite the substantially higher perioperative morbidity observed in the VV-ECMO bridge group (Fig. 4).

In contrast, postoperative AKI was strongly associated with worse long-term survival. Patients who developed AKI had significantly lower overall survival after transplantation than those without AKI (log-rank *P* < 0.0001) (supplemental Fig. 1).

## Discussion

The present study builds upon existing literature on VV-ECMO bridging to lung transplant by examining key associations with post-transplant AKI. Our goal was to expand our understanding of AKI by characterizing both short- and long-term outcomes and by offering a direct comparison of VV-ECMO bridged to non-ECMO bridged patient groups. Our results support existing literature by describing a statistically significant association between post-operative AKI and pre-operative VV-ECMO [6, 10], while also challenging previous conclusions about the impact of AKI on mortality in these settings, by having both an ECMO and non-ECMO group for comparison [5]. Importantly, we found that the increased morbidity in the VV-ECMO bridged group did not translate into a long-term survival deficit. Rather, post-operative AKI demonstrated a statistically significant, independent association with increased mortality.

Although patients bridged with VV-ECMO were significantly younger than those transplanted without ECMO bridging, this age difference did not translate into an observed survival advantage. This finding should be interpreted in the context of the markedly different clinical profiles of the two groups. The VV-ECMO group represented a more critically ill population and experienced substantially greater perioperative complexity, including more frequent preoperative dialysis, lower hemoglobin and platelet levels, bilateral transplantation, longer operative times, greater transfusion requirements, and more frequent intraoperative VA-ECMO support. These adverse clinical and operative factors may have counterbalanced the expected survival benefit associated with younger recipient age.

Furthermore, the younger age of the VV-ECMO group may reflect candidate selection, because younger patients with greater physiological reserve may be more likely to be considered suitable for ECMO bridging and subsequent transplantation. Accordingly, the absence of a statistically significant survival difference should not be interpreted as evidence of equivalence between the groups or as proof that VV-ECMO bridging has no effect on survival. Rather, our findings suggest that acceptable survival may be achievable in carefully selected VV-ECMO-bridged recipients despite their greater preoperative severity and perioperative morbidity.

An important consideration is whether the association between preoperative VV-ECMO bridging and postoperative AKI represents a direct renal effect of ECMO or reflects the greater severity and operative complexity of patients requiring bridging. Our primary multivariable model was intended to assess preoperative risk stratification and therefore included only variables available before transplantation. In this model, VV-ECMO bridging was associated with postoperative AKI. However, the association was attenuated and did not reach statistical significance after propensity score overlap weighting for measured preoperative characteristics. Similarly, the association was no longer statistically significant when bilateral transplantation, operative time, and intraoperative packed red blood cell transfusion were included in an expanded explanatory model. These findings suggest that preoperative VV-ECMO status may primarily identify recipients with greater critical illness severity who are subsequently more likely to experience a complex operative course, rather than representing a direct independent nephrotoxic exposure. Potential contributors to AKI in this population include prolonged surgery, hemodynamic instability, transfusion burden, inflammation, and exposure to additional mechanical circulatory support. In particular, the association between greater intraoperative packed red blood cell transfusion and AKI supports an important contribution from perioperative complexity. Nevertheless, the intraoperative variables may lie on the causal pathway between preoperative VV-ECMO bridging and postoperative AKI. Therefore, attenuation of the VV-ECMO estimate after adjustment for these variables does not prove that VV-ECMO has no direct renal effect. Likewise, the nonsignificant overlap-weighted estimate retained a direction toward increased AKI risk but had a wide confidence interval. The present observational data therefore cannot definitively separate direct effects, mediated effects, and residual confounding. From a clinical perspective, preoperative VV-ECMO bridging should therefore be regarded as a marker of increased perioperative renal risk rather than as an isolated contraindication to lung transplantation. Recognition of this risk before transplantation may support closer monitoring of renal function, avoidance of nephrotoxic exposures when possible, optimization of hemodynamics, and strategies to minimize operative time and transfusion burden. At the same time, the absence of a statistically significant difference in overall survival during the available follow-up suggests that appropriately selected VV-ECMO-bridged patients may still achieve acceptable post-transplant survival despite greater perioperative morbidity.

Despite the higher overall morbidity in the VV-ECMO bridge group, it was not ECMO bridging, but rather post-operative AKI, that demonstrated a significant impact on mortality post-operatively. While overall renal function declined both in patients with and without post-operative AKI in the first year post-operatively, the post-operative AKI group demonstrated a significant, sustained, poorer renal function trajectory by one year, even when accounting for differences in baseline values (supplemental Fig. 1). However, when examining overall renal function trajectories by VV-ECMO bridged and non-bridged groups, there were no significant differences at time points from one month to one year (Fig. 3). As such, closer clinical monitoring and management of early post-operative AKI in patients may be warranted, as this may be a more easily modifiable and relevant risk factor, than a patient’s need for pre-operative ECMO bridging. Furthermore, additional research stratifying AKI by etiology may aid in our understanding of ideal parameters for intra-operative and post-operative resuscitation, medication choices and titration, and optimal timing of renal replacement therapy if needed. This makes a strong case for greater collaboration with our colleagues in critical care, transplant immunology, and transplant nephrology. Our results suggest that continuing to investigate the underlying etiologies of renal dysfunction post-transplant – rather than simply attributing post-operative AKI to ECMO use alone, or assuming that ECMO use alone drives mortality – may best allow us to improve outcomes even for the sickest patients undergoing lung transplant.

Furthermore, our data addresses a key gap in current literature by evaluating results both at short-term (< 1 year) and long-term (> 5 year) time points. Overall survival was significantly lower in patients who developed post-operative AKI as compared to those who did not, and this difference was sustained from immediately post-operatively for up to 7 years of follow up (supplemental Fig. 2). However, when examining overall survival stratified by preoperative VV-ECMO bridging status, over the same long-term time interval, no significant difference in survival was identified (Fig. 4). Much of the existing literature emphasizes one-year outcomes, which raises important ethical considerations regarding long-term transplant outcomes and how we counsel patients prior to transplant. This is of particular relevance to lung transplant literature, given the relatively lower survival rate of lung transplant compared to types of other solid organ transplant, including heart transplant [27, 28]. Longer term data is relevant not only in advancing the field, but in guiding conversations with patients and their families pre-operatively, allowing providers to have more realistic, data-driven conversations about both short- and long-term outcomes.

Finally, in alignment with the newer lung Composite Allocation Score (CAS) model which broadly seeks to improve patient access to transplant [29], understanding AKI as a primary factor impacting longer term survival raises important questions regarding how we need to not only improve equitable access to lung transplant, but also promote positive outcomes among our sickest patients. Many of the factors that we found to be independently associated with post-operative AKI, including hypertension and chronic kidney disease, are known to disproportionately impact lower socioeconomic status and racially diverse patients [30, 31]. While no lung transplant literature to date has examined post-operative AKI in the context of known healthcare disparities, a recent meta-analysis described socioeconomic status as an independent predictor of mortality among patients with AKI [32]. An attention not only to access, but to outcomes, among minoritized populations should remain a key area of focus for future lung transplant literature.

### Limitations

This study has several limitations. First, it was a retrospective, single-center study with a substantial imbalance in sample size between the VV-ECMO and non-ECMO groups. Only 46 patients were bridged with VV-ECMO, compared with 397 patients who underwent transplantation without ECMO bridging, which limited statistical power and the precision of effect estimates.

Second, the two groups differed substantially in both baseline characteristics and perioperative management. Patients bridged with VV-ECMO were younger, had different underlying lung disease distributions and waiting-list durations, and underwent more complex operative courses, including more frequent bilateral transplantation, greater transfusion requirements, longer operative times, and more frequent intraoperative VA-ECMO support. Although clinically relevant variables were included in the multivariable analyses, residual confounding related to illness severity, recipient selection, operative complexity, and other unmeasured factors cannot be excluded.

In particular, the younger age of the VV-ECMO group might have been expected to confer a survival advantage. However, this potential benefit may have been offset by greater preoperative critical illness and perioperative morbidity. Younger age may also reflect selection of patients with sufficient physiological reserve to undergo ECMO bridging and transplantation. Therefore, the absence of a statistically significant survival difference should not be interpreted as demonstrating equivalence between groups or establishing that VV-ECMO bridging has no effect on survival. The assessment of postoperative AKI in patients who had received renal replacement therapy before transplantation was inherently challenging because serum creatinine may be affected by dialysis, fluid balance, reduced muscle mass, and critical illness. Although preoperative dialysis alone was not used to define postoperative AKI, some degree of misclassification cannot be excluded. Detailed information regarding the timing, modality, intensity, and indication for preoperative renal replacement therapy was not uniformly available because of the retrospective design. Renal function measurements at the time of transplant listing and before VV-ECMO initiation were not systematically available. Consequently, the pretransplant serum creatinine and eGFR values used in this study may not reflect stable intrinsic renal function before critical illness. Creatinine-based renal indices may overestimate renal function in critically ill patients because of reduced muscle mass, malnutrition, fluid accumulation, and altered creatinine kinetics. This limitation may have been particularly relevant in the VV-ECMO group and may have affected both the comparison of pretransplant renal function between groups and the estimated association between serum creatinine and postoperative AKI. It will require future research to examine these intricacies in order to best guide actionable changes from a clinical management perspective.

## Supplementary Information

Below is the link to the electronic supplementary material.

Supplementary file1 (docx 441 KB)

## Data Availability

The data presented in this study are available on reasonable request by a qualified investigator for three years after the date of publication from the corresponding author.
