# Supplementary material for: Impact of preoperative venovenous extracorporeal membrane oxygenation bridging on postoperative acute kidney injury after lung transplantation
Source: J Artif Organs. 2026 Aug 17;29(3):56. doi: 10.1007/s10047-026-01585-4 (PMC13481578; doi:10.1007/s10047-026-01585-4)
Supplement: Supplementary file 1 [file 10047_2026_1585_MOESM1_ESM.docx]

**Supplemental Table1**

| **Supplemental Table 1. Multivariable logistic regression analysis of predictors of postoperative acute kidney injury including intraoperative variables** | | | |  |
| --- | --- | --- | --- | --- |
|  | **Multivariate** | | |  |
| Variable | OR | 95% CI | P value |  |
| **Pre-operative Characteristics** |  |  |  |  |
| Age, years | 1.00 | 0.98-1.02 | 0.89 |  |
| Sex |  |  |  |  |
| Male | 0.99 | 0.48-1.22 | 0.27 |  |
| BMI | 1.04 | 0.99-1.09 | 0.12 |  |
| Hypertension | 1.96 | 1.25-3.15 | 0.004 |  |
| Diabetes | 1.78 | 0.98-2.50 | 0.06 |  |
| Preoperative VV-ECMO | 2.60 | 0.43-3.40 | 0.73 |  |
| Etiology |  |  |  |  |
| ILD | 0.76 | 0.50-1.68 | 0.76 |  |
| COPD | 0.67 | 0.38-1.64 | 0.52 |  |
| PAH | 0.83 | 0.37-1.92 | 0.68 |  |
| Laboratory |  |  |  |  |
| Hemoglobin, g/dL | 0.89 | 0.81-1.01 | 0.08 |  |
| Creatinine, mg/dL | 7.10 | 6.23-49.70 | <0.001 |  |
| **Intra-operative outcomes** |  |  |  |  |
| Bilateral | 1.62 | 0.93-2.86 | 0.09 |  |
| Operative time (hours) | 0.90 | 0.77-1.05 | 0.18 |  |
| Intra-op blood transfusion; pRBC | 1.15 | 1.04-1.29 | 0.01 |  |
| Odds ratios (OR) and 95% confidence intervals (CIs) were estimated using a multivariable logistic regression model for postoperative AKI that included age, sex, smoking history, hypertension, diabetes, preoperative VV-ECMO bridging, underlying lung disease etiology, hemoglobin, serum creatinine, bilateral transplantation, operative time, and intraoperative packed red blood cell transfusion. Continuous variables were modeled per 1-unit increase. Female sex, no smoking history, no hypertension, no diabetes, no preoperative VV-ECMO bridging, other underlying lung disease etiologies, and single-lung transplantation were used as the reference categories. VV-ECMO, venovenous extracorporeal membrane oxygenation; AKI, acute kidney injury; ILD, interstitial lung disease; COPD, chronic obstructive pulmonary disease; PAH, pulmonary arterial hypertension; pRBC, packed red blood cells; aOR, adjusted odds ratio; CI, confidence interval. | | | |  |
|  |  |  |  |  |
|  |  |  |  |  |
|  |  |  |  |  |
|  |  |  |  |  |
|  |  |  |  |  |
|  |  |  |  |  |

**Supplemental Table2**

| Supplemental Table 2. Covariate balance before and after propensity score overlap weighting | | |  |
| --- | --- | --- | --- |
| Variable | Absolute SMD before weighting | Absolute SMD after weighting |  |
| Age | 1.03 | 0.00 |  |
| Male sex | 0.13 | 0.00 |  |
| Body mass index | 0.07 | 0.00 |  |
| Smoking history | 0.70 | 0.00 |  |
| Hypertension | 0.31 | 0.00 |  |
| Diabetes | 0.12 | 0.00 |  |
| Chronic kidney disease | 0.13 | 0.00 |  |
| Preoperative dialysis | 0.40 | 0.00 |  |
| Waiting-list duration | 0.23 | 0.00 |  |
| Etiology: Others | 1.25 | 0.00 |  |
| Etiology: ILD | 0.58 | 0.00 |  |
| Etiology: COPD | 0.74 | 0.00 |  |
| Etiology: PAH | 0.14 | 0.00 |  |
| Hemoglobin | 2.39 | 0.00 |  |
| Platelet count | 1.15 | 0.00 |  |
| Serum creatinine | 0.74 | 0.00 |  |
| Positive PRA | 0.39 | 0.00 |  |
| Covariate balance was assessed using absolute standardized mean differences before and after propensity score overlap weighting. An absolute standardized mean difference of less than 0.10 was considered indicative of adequate balance. All measured preoperative covariates achieved adequate balance after overlap weighting. SMD, standardized mean difference; ILD, interstitial lung disease; COPD, chronic obstructive pulmonary disease; PAH, pulmonary arterial hypertension; PRA, panel reactive antibody. | | |  |
|  |  |  |  |
|  |  |  |  |
|  |  |  |  |

**Supplemental Table 3**

| Supplemental Table 3. Propensity score overlap-weighted analysis of the association between preoperative VV-ECMO bridging and postoperative acute kidney injury | | | | |  |
| --- | --- | --- | --- | --- | --- |
| **Outcome** | **Exposure** | **Weighted OR** | **95% CI** | **P value** |  |
| Postoperative AKI | Preoperative VV-ECMO bridging | 2.34 | 0.87-6.31 | 0.093 |  |
| The propensity score was estimated using age, sex, body mass index, smoking history, hypertension, diabetes, chronic kidney disease, preoperative dialysis, waiting-list duration, underlying lung disease etiology, hemoglobin, platelet count, serum creatinine, and panel reactive antibody status. Overlap weighting was used to estimate the association between preoperative VV-ECMO bridging and postoperative AKI. Weighted odds ratios and 95% confidence intervals were estimated using weighted logistic regression with robust standard errors. VV-ECMO, venovenous extracorporeal membrane oxygenation; AKI, acute kidney injury; OR, odds ratio; CI, confidence interval. | | | | |  |
|  |  |  |  |  |  |
|  |  |  |  |  |  |
|  |  |  |  |  |  |

**Supplemental Figure 1**

**Supplemental Figure 2**
